# Adherence to Internet-Based Mobile-Supported Stress Management: A Pooled Analysis of Individual Participant Data From Three Randomized Controlled Trials

**DOI:** 10.2196/jmir.4493

**Published:** 2016-06-29

**Authors:** Anna-Carlotta Zarski, Dirk Lehr, Matthias Berking, Heleen Riper, Pim Cuijpers, David Daniel Ebert

**Affiliations:** ^1^Department of Clinical Psychology and PsychotherapyFriedrich-Alexander University Erlangen-NurembergErlangenGermany; ^2^Innovation IncubatorDivision of Online Health TrainingLeuphana University LueneburgLuenburgGermany; ^3^Institute for PsychologyDepartment of Health Psychology and Applied Biological PsychologyLeuphana University LuenburgLueneburgGermany; ^4^Department of Clinical PsychologyVU University AmsterdamAmsterdamNetherlands; ^5^Telepsychiatric CentreUniversity of Southern DenmarkOdenseDenmark

**Keywords:** guidance, treatment adherence, predictors, Internet intervention, work-related stress, stress management

## Abstract

**Background:**

Nonadherence to treatment is a prevalent issue in Internet interventions. Guidance from health care professionals has been found to increase treatment adherence rates in Internet interventions for a range of physical and mental disorders. Evaluating different guidance formats of varying intensity is important, particularly with respect to improvement of effectiveness and cost-effectiveness. Identifying predictors of nonadherence allows for the opportunity to better adapt Internet interventions to the needs of participants especially at risk for discontinuing treatment.

**Objective:**

The goal of this study was to investigate the influence of different guidance formats (content-focused guidance, adherence-focused guidance, and administrative guidance) on adherence and to identify predictors of nonadherence in an Internet-based mobile-supported stress management intervention (ie, GET.ON Stress) for employees.

**Methods:**

The data from the groups who received the intervention were pooled from three randomized controlled trials (RCTs) that evaluated the efficacy of the same Internet-based mobile-supported stress management intervention (N=395). The RCTs only differed in terms of the guidance format (content-focused guidance vs waitlist control, adherence-focused guidance vs waitlist control, administrative guidance vs waitlist control). Adherence was defined by the number of completed treatment modules (0-7). An ANOVA was performed to compare the adherence rates from the different guidance formats. Multiple hierarchical linear regression analysis was conducted to evaluate predictors of nonadherence, which included gender, age, education, symptom-related factors, and hope for improvement.

**Results:**

In all, 70.5% (93/132) of the content-focused guidance sample, 68.9% (91/132) of the adherence-focused guidance sample, and 42.0% (55/131) of the participants in the administrative guidance sample completed all treatment modules. Guidance had a significant effect on treatment adherence (*F*_2,392_=11.64, *P*<.001; ω^2^=.05). Participants in the content-focused guidance (mean 5.70, SD 2.32) and adherence-focused guidance samples (mean 5.58, SD 2.33) completed significantly more modules than participants in the administrative guidance sample (mean 4.36, SD 2.78; *t*_223_=4.53, *P*<.001; *r*=.29). Content-focused guidance was not significantly associated with higher adherence compared to adherence-focused guidance (*t*_262_=0.42, *P*=.67; *r*=.03). The effect size of *r*=.03 (95% CI –0.09 to 0.15) did not pass the equivalence margin of *r*=.20 and the upper bound of the 95% CI lay below the predefined margin, indicating equivalence between adherence-focused guidance and content-focused guidance. Beyond the influence of guidance, none of the predictors significantly predicted nonadherence.

**Conclusions:**

Guidance has been shown to be an influential factor in promoting adherence to an Internet-based mobile-supported stress management intervention. Adherence-focused guidance, which included email reminders and feedback on demand, was equivalent to content-focused guidance with regular feedback while requiring only approximately a quarter of the coaching resources. This could be a promising discovery in terms of cost-effectiveness. However, even after considering guidance, sociodemographic, and symptom-related characteristics, most interindividual differences in nonadherence remain unexplained.

**Clinical Trial:**

DRKS00004749; http://drks-neu.uniklinik-freiburg.de/drks_web/navigate.do?navigationId=trial.HTML&TRIAL _ID=DRKS00004749 (Archived by WebCite at http://www.webcitation.org/6QiDk9Zn8); DRKS00005112; http://drks-neu.uniklinik-freiburg. de/drks_web/navigate.do?navigationId=trial.HTML&TRIAL_ID=DRKS00005112 (Archived by WebCite at http://www.webcitation.org/6QiDysvev); DRKS00005384; http://drks-neu.uniklinik-freiburg.de/ drks_web/navigate.do?navigationId=trial.HTML&TRIAL_ID=DRKS00005384 (Archived by WebCite at http://www.webcitation.org/6QiE0xcpE)

## Introduction

Many participants of Internet-based programs do not begin the interventions after registration or are nonadherent and quit the intervention prematurely against recommendations in both study and routine care settings [1-6]. Treatment adherence can be defined as the extent to which individuals experience the intervention content [7]. Low treatment adherence is a major concern because it has been associated with reduced efficacy of Internet interventions for physical and mental disorders and health promotion programs [8-13].

Occupational stress is associated with an increased risk for common mental disorders in the long term [14]. Internet-based stress management interventions for employees show promising effects in reducing stress and related health problems such as depression [15-19]. Nevertheless, low treatment adherence is a serious problem that adversely impacts the efficacy of Internet-based antistress programs [20-22].

In order to improve the clinical effects of Internet interventions, it is important to identify and evaluate factors associated with adherence [23]. Treatment factors, in particular human guidance, may be especially important [24-26]. Guided treatments have been shown to result in higher adherence rates (56% to 81%) than unguided treatments (26% to 69%) [27-31]. Treatment adherence rates of guided interventions have often been found to be comparable to those of face-to-face interventions [32,33]. However, guidance from a health care professional is expensive and a limited resource. Thus, it is crucial to investigate the effects of different guidance formats to identify the level and type of guidance necessary to achieve acceptable treatment adherence.

Guidance in Internet interventions is often classified according to the amount of required coaching or therapist time [34]. However, guidance also differs regarding objectives and content. Accordingly, at least four different guidance formats can be distinguished: (1) unguided interventions, completely self-administered by the user; (2) administrative guidance, providing technical support in case of computer and Internet platform-related problems and dispensing relevant information throughout the course of the treatment; (3) adherence-focused guidance, with adherence monitoring including reminders by email or telephone; and (4) content-focused guidance, personalized written feedback by coaches for completed treatment modules and adherence monitoring.

Content-focused guidance in Internet interventions has been found to be associated with higher levels of treatment completion (72%) compared to administrative guidance (62%) or unguided interventions (26%) in a meta-analysis [31]. Adherence-focused guidance also seems promising in fostering treatment completion with the added benefit of keeping coaching demands—in terms of time spent per participant—to a minimum [26,35-39].

However, whether adherence-focused guidance results in comparable adherence rates to content-focused guidance formats remains unclear. Comparisons of adherence-focused guidance to content-focused guidance are currently limited regarding treatment adherence in Internet interventions.

Mohr and colleagues [25] introduced the notion of “supportive accountability” to explain the relationship between human guidance and treatment adherence in Internet interventions. This model assumes that human support in Internet interventions enhances adherence by allowing the patient to foster a commitment toward an eCoach, who is perceived as being trustworthy, benevolent, and professionally knowledgeable. The eCoach is responsible for accompanying the participants through the program, showing interest in their training processes, and offering support. In this respect, adherence monitoring in addition to reminders in case of nonadherence must be embedded in a benevolent context, with the aim of supporting adherence instead of surveillance of module completion. The expectations placed on the participant by the therapy program, to be sustained on a regular basis throughout the treatment, should be transparent and reasonable. Hence, the participant knows what is to be expected and can be involved in determining these expectations. Both in adherence- and content-focused guidance, the eCoach communicates the requirements for successful participation in the intervention and the associated expectations placed on the participants. By contrast, in unguided or administrative-guided interventions, participants only receive general recommendations on program use.

Based on the supportive accountability model [25], we assumed that the introduction of an eCoach who offers support in program completion is important for creating an adherence-promoting relationship with the participant. For this reason, we developed the concept of adherence-focused guidance, which is comprised of not only adherence monitoring, but also the opportunity to receive content feedback on demand [40]. At the beginning of the training, the eCoach invites the participant to contact them in case of content-related questions and any desired feedback for completed treatment modules. Feedback on demand is expected to emphasize the supporting role of the eCoach in the training process and thereby promote the participant’s adherence. Assuming that accountability is the essential factor in coaching that keeps participants involved in training, we hypothesized that both adherence-focused guidance and content-focused guidance are superior to administrative guidance with regard to adherence rates. Adherence-focused guidance was expected to be equivalent to content-focused guidance for adherence rates.

Apart from treatment factors, knowledge of user characteristics related to nonadherence helps to identify individuals who are at risk of discontinuing treatment and might need additional support. According to the behavior change model for Internet interventions by Ritterband and colleagues [41], user characteristics can be classified into disease-related factors, demographics, traits, cognitive factors, beliefs and attitudes, physiological factors, and skills.

Demographic and disease-specific user characteristics were of particular interest in past adherence research in the field of Internet interventions. Low education level [1,42-44], male gender [1,30,42,45], or being unmarried or single [43,46-48] have been found to be associated with lower treatment adherence across different mental and physical health interventions. Younger age has also been shown to be related to nonadherence in the majority of studies [37,42,45,49]. Only a small number of studies found older individuals to be at a higher risk for nonadherence [1,2,7]. However, some studies found no significant association between any sociodemographic variables and treatment adherence [7,50,51].

High symptom severity at baseline is also frequently linked to lower treatment adherence [7,47,52,53]. But, the relationship between baseline depressive symptoms and treatment adherence seems inconsistent. Both higher [7,54,55] and lower [1,3,42] baseline depression scores were found to be associated with lower treatment adherence or no significant association was found [30,50,56,57]. Lower treatment expectations have been found to be related to nonadherence in Internet interventions [56,58,59].

Only a few studies to date have investigated predictors of adherence in Internet intervention and they showed conflicting results [60]. For this reason, we used an exploratory approach including potential predictors based on (1) results of previous studies investigating adherence predictors in Internet interventions [1,7,42-44] and (2) theoretical assumptions due to intervention characteristics. The final list of potential predictors investigated in the present study included: (1) demographics: gender, education level, and age; (2) symptom-related factors: stress, depression, and emotional exhaustion; and (3) variables concerning beliefs and attitudes: hope for improvement.

To the best of our knowledge, no research to date has explored the influence of different guidance formats and user characteristics on treatment adherence to an Internet intervention in stressed employees.

The current study aimed to (1) report adherence rates from a newly developed Internet-based mobile-supported stress management intervention (ie, GET.ON Stress) and (2) investigate the role of different guidance formats (content-focused guidance, adherence-focused guidance, and administrative guidance) on adherence. Further goals of the study were to (3) identify user characteristics predictive of treatment nonadherence over and above the guidance formats and (4) analyze differential predictor effects as a function of guidance formats.

We hypothesized that (1) treatment adherence rates would be greater for content-focused guidance and adherence-focused guidance compared to administrative guidance, and (2) adherence-focused guidance is equivalent to content-focused guidance in terms of adherence rates. User characteristics that contribute to treatment nonadherence apart from guidance formats and differential effects of predictors as a function of guidance formats were analyzed exploratively. In this study, treatment adherence is operationalized by the number of completed treatment modules.

## Methods

Data for this analysis were drawn from three randomized controlled trials (RCTs) evaluating the same Internet-based mobile-supported stress management intervention (GET.ON Stress [61-64]) under varying guidance conditions (study 1: content-focused guidance vs waitlist control; study 2: adherence-focused guidance vs waitlist control; study 3: administrative guidance vs waitlist control). Details of the study design for study 1 have been described in a published study protocol [61]. All three trials employed the same design and procedures apart from the guidance format, allowing for the pooling of the data from the three studies.

### Sample

The analyses in this study were based solely on the intervention group samples of the N=395 participants who received the same Internet-based mobile-supported stress management intervention (study 1: n=132; study 2: n=132; study 3: n=131). Participants in the waitlist control condition were not included in the analyses because they did not receive access to the training until 6 months after randomization. All three studies included (1) currently employed workers, (2) older than age 18 years, (3) with scores ≥22 on the Perceived Stress Scale (PSS-10) [65], (4) who self-reported having Internet access, (5) sufficient skills in reading and writing German, and (6) who were willing to give informed consent. Participants were excluded when (1) they self-reported having been diagnosed with psychoses or dissociative symptoms in the past or (2) showed a notable suicidal risk as indicated by a score of greater than 1 on item 9 (“I feel I would be better off dead”) on the Beck Depression Inventory (BDI) [66].

### Intervention

The Internet-based mobile-supported stress management intervention GET.ON Stress is based on two main components: problem solving [16,67-69] and emotion regulation [70,71]. A detailed description can be found elsewhere [61]. The intervention consists of seven modules composed of psychoeducation (module 1), problem solving (modules 2-3), emotion regulation (modules 4-6), planning for the future (module 7), and an optional booster session. Additionally, participants are offered eight units that are integrated in modules 2 to 6 that can be opted for based on individual needs or preference. These units are directed at time management, rumination and worrying, psychological detachment from work, sleep hygiene, rhythm and regularity of sleeping habits, nutrition and exercise, organization of breaks during work, and social support. Each module can be completed in approximately 45 to 60 minutes. Participants were advised to do at least one and a maximum of two modules per week. Consequently, the intervention took approximately 4 to 7 weeks (not including the booster session offered 4 weeks after completion of the intervention). Lessons were in the format of text, exercises, and testimonials, and included interactive elements such as audio and video clips. Participants were encouraged to keep a daily online stress diary. One strong focus of the intervention lay in transfer tasks (homework assignments) to integrate the newly acquired strategies and techniques into daily life. The training was adaptive because the content is tailored to the specific needs of the individual participants by continuously asking them to choose among various response options. Using responsive Web design, participants could use the program on a computer, tablet, or mobile phone. An integrated read-aloud function allowed participants to follow narrated lessons. If desired, participants could receive automatic motivational text messages and small exercises on their mobile phones. These messages had the purpose of supporting the participant in transferring the exercises of the training into their daily lives (eg, short relaxation exercises). The participants had the opportunity to choose between “light coach” (one text message every other day) or “intensive coach” (two to three text messages every day) options.

### Content-Focused Guided Internet-Based Mobile- Supported Stress Management Intervention

Participants in the content-focused guidance condition received personalized written feedback from an eCoach on the exercises they had completed in each module within 48 hours. The eCoaches were psychologists and trained Master’s-level psychology students who followed guidelines about the feedback process that were defined according to the standardized manual for the intervention. The eCoaches were advised to not spend more than 30 minutes on feedback on a given completed module. The eCoaches sent reminders when the participants did not complete a module within 7 days. In total, the eCoaches sent 365 reminders, corresponding to a mean 2.77 reminders per participant (range 0-11, SD 2.41). The time required for coaching totaled up to 4 hours per participant.

### Adherence-Focused Guided Internet-Based Mobile- Supported Stress Management Intervention

Participants of the adherence-focused guidance condition were also supported by an eCoach. The guidance manual was based on our developed adherence-focused guidance concept as outlined in the Introduction [40]. The eCoaches were trained psychologists who followed guidelines about the feedback process that were defined according to the standardized manual for the intervention. Adherence-focused guidance consisted of adherence monitoring and feedback on demand. Adherence monitoring included regularly checking module completion and sending reminders in case the participant did not complete at least one module within 7 days. In total, the eCoaches sent 463 reminders, corresponding to a mean 3.51 reminders per participant (range 0-13, SD 1.98). Feedback on demand included offering participants the opportunity to contact the eCoach and receive individual support or feedback within 24 hours. In contrast to the content-focused guidance concept, eCoach guidance only took place at the initiative of the participants to minimize the costs. There were only requests for a mean 8 content feedbacks by all participants (range 0-5, SD 0.46), corresponding to 0.06 feedback demands per participant. Thus, most of the time spent per participant was related to checking adherence to the intervention and providing reminders in case of nonadherence. The time required for coaching, including all reminders and feedback by request, added up to 1 hour per participant.

### Administrative-Guided Internet-Based Mobile- Supported Stress Management Intervention

Participants in the administrative guidance condition were provided with contact information for the study administration team during the study period, which addressed such things as the completion of questionnaires, but they were not supported by an eCoach. They were provided with an email address to use in case of any technical problems.

### Measures

#### Adherence Measures

The number of completed treatment modules in the Internet-based mobile-supported stress management intervention, which ranged from 0 to 7, was the primary outcome measure in this study and was assessed by the system that provided the intervention. Module completion was defined by completion of the last page of a module. To arrive on the last page, participants were required to complete all the previous writing tasks. A module completion score of 0 could either mean that the participant did not start the intervention or did not finish the first module. Each module took approximately 45 to 60 minutes for completion.

#### Predictor Measures

The following variables, assessed at baseline before the start of the program, were evaluated as potential predictors of nonadherence: sociodemographic factors (gender [male/female], age [years], level of education [low, middle, high]), symptom severity factors (perceived stress, depressive symptoms, emotional exhaustion), and hope for improvement (confidence in treatment efficacy).

Perceived stress at baseline relating to the past week was examined with the German version of the 10-item Perceived Stress Scale (PSS-10) [65,72]. This self-report instrument uses a 5-point Likert-type scale that ranges from 0=“never” to 4=“very often.” A Cronbach alpha of .77 indicated acceptable internal consistency of the PSS-10 in this study.

Baseline depression symptom severity was measured with the German version of the Center for Epidemiological Studies Depression Scale (CES-D) [73,74]. This frequently used self-report instrument consists of 15 items that are answered on a 4-point Likert-type scale and refer to the previous week. Total scores range from 0 to 60. In this study, internal consistency was good (Cronbach alpha=.88).

To measure emotional exhaustion, the basic stress dimension of burnout, the German version of the Maslach Burnout Inventory was utilized (MBI-GS-D) [75,76]. This commonly used self-report instrument consists of five items and uses a 6-point Likert-type scale anchored by 1=“never” and 6=“very often.” In this study, internal consistency was acceptable (Cronbach alpha=.79).

Hope for improvement (confidence in treatment efficacy) was measured using the homonymous subscale of the German Patient Questionnaire on Therapy Expectation and Evaluation (PATHEV) [77] adapted to the online training context. The items are rated on a 5-point Likert-type scale. The hope for improvement subscale showed acceptable internal consistency (Cronbach alpha=.79) in this study.

### Analysis

An ANOVA was conducted to compare the treatment adherence rates between the three guidance formats with guidance as the independent variable and adherence as the dependent variable [78]. The power analysis revealed that with the given sample size (N=395), small effects (*ω*^2^*=* 0.03) with a power (1-beta) of 80% and alpha=.05 would have been detected. Omega squared (*ω*^2^) was used as the measure for effect size of the ANOVA overall effect with values of 0.01, 0.06, and 0.14 representing small, medium, and large effects, respectively [79].

In planned contrasts, the superiority of the content-focused guidance and adherence-focused guidance over administrative guidance as well as equivalence of adherence-focused guidance and content-focused guidance was assessed. The effect sizes for the planned comparisons were described by *r* with values of .1, .3, and .5 indicating small, medium, and large effects, respectively [80]. In order to investigate the hypothesis that adherence-focused guidance is equivalent to content-focused guidance, a confidence interval approach was used on the effect size of the difference between adherence-focused guidance and content-focused guidance with a two-sided .05 level of significance [81]. The equivalence margin was set a priori at *r=*.20 corresponding to the smallest value that would present a relevant effect [82]. The upper bound of the 95% CI for the effect size was compared with the predefined equivalence margin of *r*=.20 and had to be below the margin to show equivalence, with a significance level of .05. Hierarchical multiple linear regression analysis was performed on the combined sample to explore potential predictors of nonadherence in addition to guidance formats with gender, age, education level, stress level, depression, emotional exhaustion, and hope for improvement as the independent variables, and adherence as the dependent variable [78]. In this model, sociodemographic variables (gender, age, and education level) were entered in the first step, followed by baseline symptom-related factors (stress level, depression, and emotional exhaustion) and hope for improvement in the second step, and guidance (content-focused guidance vs administrative guidance, adherence-focused guidance vs administrative guidance) in the third step. In the final step, the interactions between the guidance formats and the other predictor variables were added to the model to explore potentially differential predictor effects as a factor of guidance formats.

All continuous predictors were group mean centered. The power analysis revealed that with the given sample size (N=395), small effects (*r*=.20) with a power (1–beta) of 80% and Cronbach alpha=.05 would have been detected.

One participant had missing values in the depression level and the hope of improvement variable for which data were imputed using a Markov chain Monte Carlo multivariate imputation algorithm (missing data module in SPSS version 22) with 100 estimations per missing value.

In order to test the robustness of our results, we applied sensitivity analyses. We used (1) a more conservative outcome by defining modules as completed only when finished within 12 weeks and (2) Kaplan-Meier survival curves to compare the adherence rates between the three different guidance formats. All analyses were performed using SPSS version 22. Directed hypotheses were tested with a one-tailed test and nondirected hypotheses with a two-tailed test.

## Results

### Descriptive Statistics

In total, 395 participants were included in the analysis. Baseline characteristics of the study population are presented in Table 1. There was a significant difference in the gender ratio and the education level between the three studies. The adherence-focused guidance sample showed a significantly lower percentage of male participants (13.6%, 18/132) compared to the administrative guidance (26.0%, 34/131) and content-focused guidance samples (26.5%, 35/132). The content-focused guidance sample had a significantly higher education level (64.4%, 85/132) compared to the administrative guidance (56.5%, 74/131) and adherence-focused guidance samples (52.3%, 69/132). However, gender and education level were not associated with treatment adherence in the explorative analysis and, thus, not accounted for in subsequent analyses.

**Table 1:** Baseline characteristics of the study population (N=395).

Characteristic		Administrative guidance (n=131)	Adherence-focused guidance (n=132)	Content-focused guidance (n=132)	*P*
Age (years), mean (SD)		41.2 (9.4)	42.6 (9.5)	42.4 (10.7)	.51
**Gender, n (%)**					.02
	Female	97 (74.1)	113 (85.6)	97 (73.5)	
	Male	34 (26.0)	18 (13.6)	35 (26.5)	
	Other		1 (0.8)		
**Ethnicity, n (%)**					.98
	Caucasian	107 (81.7)	108 (81.8)	110 (83.3)	
	Asian	1 (0.8)	1 (0.8)	0	
	Not reported	23 (17.6)	23 (17.4)	22 (16.7)	
**Marital status, n (%)**					.96
	Unmarried	40 (30.5)	39 (29.6)	43 (32.6)	
	Married	65 (49.6)	62 (47.0)	63 (47.7)	
	Cohabited	16 (12.2)	18 (13.6)	17 (12.9)	
	Separated	9 (6.9)	13 (9.9)	8 (6.1)	
	Widowed	1 (0.8)	0	1 (0.8)	
**Education, n (%)**					.01
	Low	0	1 (0.8)	5 (3.8)	
	Middle	57 (43.5)	62 (47.0)	42 (31.8)	
	High	74 (56.5)	69 (52.3)	85 (64.4)	
**Gross annual income (in Euro), n (%)**					.34
	Low	39 (29.8)	41 (31.1)	35 (26.5)	
	Middle	40 (30.5)	33 (25.0)	26 (19.7)	
	High	45 (34.4)	49 (37.1)	59 (44.7)	
	Not reported	7 (5.3)	9 (6.8)	12 (9.1)	
**Employment status, n (%)**					.42
	Permanent	104 (79.4)	107 (81.1)	110 (83.3)	
	Temporary	19 (14.5)	14 (10.6)	11 (8.3)	
	Self-employed	6 (4.6)	9 (6.8)	11 (8.3)	
	Other	2 (1.5)	2 (1.5)	0	
**Experience with health-related programs, n (%)**					.82
	Yes	14 (10.7)	17 (12.9)	17 (12.9)	
	No	117 (89.3)	115 (87.1)	115 (87.1)	
**Experience with face-to-face psychotherapy, n (%)**					.32
	Yes	47 (35.9)	46 (34.9)	57 (43.2)	
	No	84 (64.1)	86 (65.2)	75 (56.8)	
Stress, mean (SD)		25.7 (5.0)	25.2 (4.6)	25.9 (3.9)	.44
Depression, mean (SD)		25.1^a^ (9.3)	23.2 (9.3)	23.3 (8.5)	.15
Emotional exhaustion, mean (SD)		4.8 (0.8)	4.7 (0.8)	4.7 (0.7)	.96
Hope of improvement, mean (SD)		3.7^a^ (0.6)	3.6 (0.6)	3.7 (0.7)	.27

^a^ Due to missing data, the means refer to a subsample with n=130 in this group.

### Adherence Rates Between Guidance Formats

**Figure 1:**
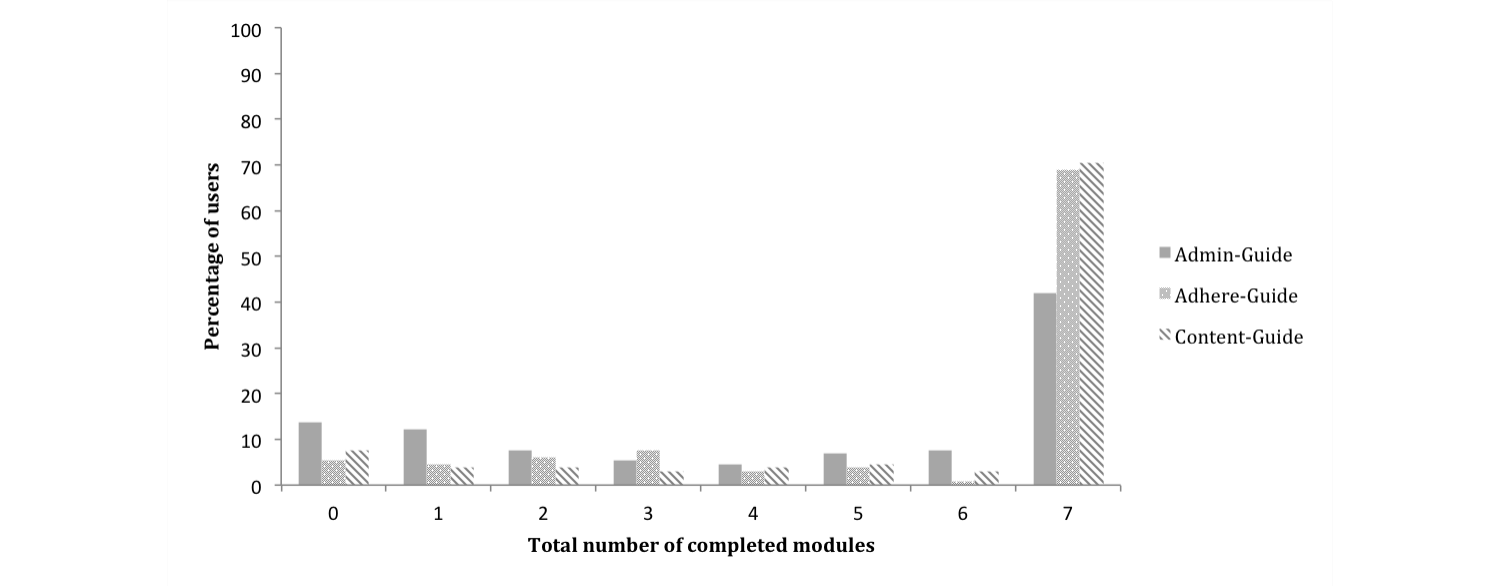
Total number of completed modules per participant.

**Figure 2:**
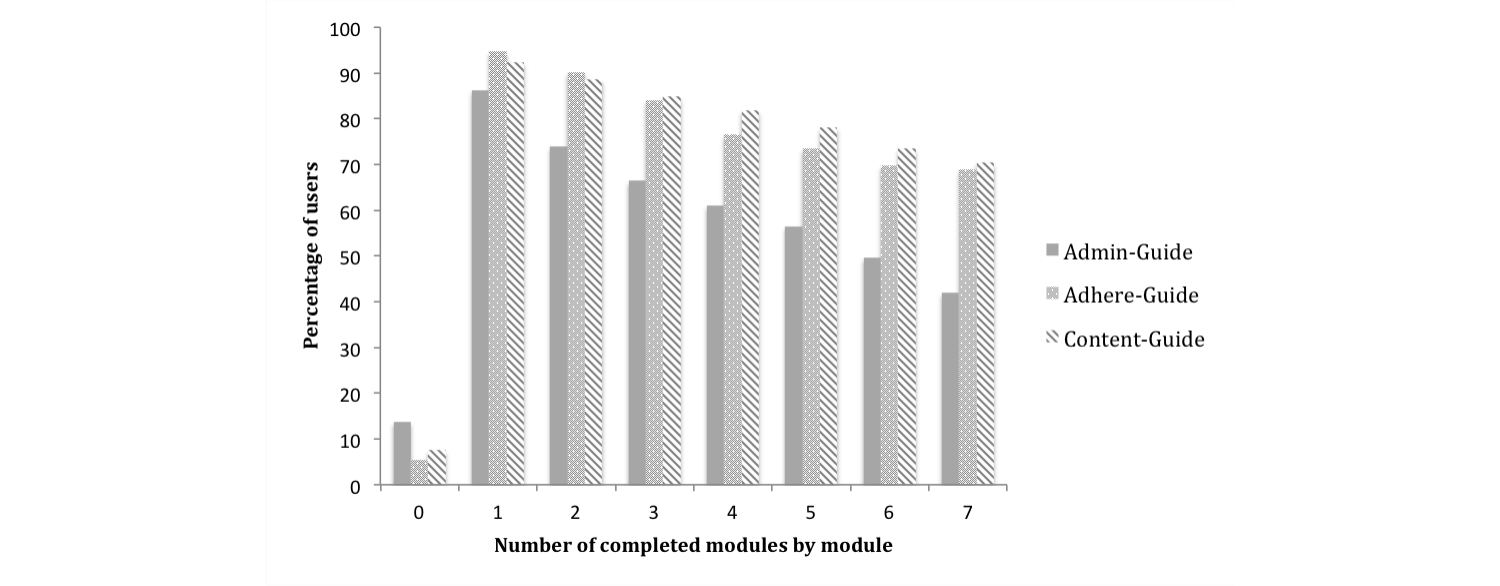
Number of completed modules by module.

Figures 1 and 2 depict the treatment adherence rates for all three samples. Figure 1 shows the total number of completed modules per participant and Figure 2 depicts the number of completed modules by module. In the administrative guidance sample, 13.7% (18/131) of the participants did not start the intervention, and 42.0% (55/131) completed all seven modules with a mean of 4.4 completed modules (SD 2.8, range 0-7). In the group that received adherence-focused guidance, 5.3% (7/132) of the participants did not start the intervention and 68.9% (91/132) completed all modules with a mean number of 5.6 completed modules (SD 2.3, range 0-7). In the content-focused guidance sample, 7.6% (10/132) of the participants did not start the intervention and 70.5% (93/132) completed all modules with a mean number of 5.7 completed modules (SD 2.3, range 0-7).

### Guidance Formats on Adherence

As expected, there was a significant effect of guidance on treatment adherence (*F*_2,392_=11.64, *P*<.001; *ω*^2^=.05). Planned comparisons revealed that (1) participants in the content-focused guidance (mean 5.7, SD 2.3) and adherence-focused guidance samples (mean 5.6, SD 2.3) completed significantly more modules than the participants in the administrative guidance sample (mean 4.4, SD 2.8; *t*_223_=4.53, *P<*.001; *r*=.29) and (2) there was no significant difference between the content-focused guidance and adherence-focused guidance groups in treatment adherence (*t*_262_=0.42, *P=*.67; *r*=.03). The effect size *r*=.03 (95% CI –0.09 to 0.15) did not cross the equivalence margin of *r=*.20. Likewise, the upper bound of the two-sided 95% confidence interval lay below the predefined margin, indicating that adherence-focused guidance was equivalent to content-focused guidance. Hence, we conclude that adherence-focused guidance is not associated with relevant lower treatment adherence compared to content-focused guidance.

Using a more conservative outcome by defining modules as completed only when finished within 12 weeks did not result in any different conclusions (results not shown). Similarly, conducting survival analysis according to Kaplan-Meier, we derived comparable results. The survival distribution for administrative guidance was significantly different from the survival distribution of adherence-focused guidance (χ^2^_2_=19.0, *P*<.001) and significantly different from the survival distribution of content-focused guidance (χ^2^_2_=21.7, *P*<.001). No significant difference was found between the survival distributions of adherence-focused guidance and content-focused guidance (χ^2^_2_=0.1, *P=*.77).

### User Characteristics Predictive of Nonadherence

Table 2 shows the results of the imputed hierarchical multiple linear regression analysis examining predictors of treatment nonadherence over and above the guidance format. Due to missing values, the depression level variable and the hope of improvement variable of one participant had to be imputed. Entering the sociodemographic variables (gender, age, and education level) as a first step into the model did not significantly explain variance (*R*^2^=.02 *, P=*.09). Entering the symptom-related factors (stress level, depression, emotional exhaustion) and hope for improvement as a second block into the model resulted in significantly more explained variance (∆ *R*^2^=.02 *, P=*.046). Stress level and depression were predictive of treatment nonadherence in this step (*t*_385_=2.00, *P=*.046; *t*_385_=–2.10, *P=*.04). However, none of these factors was predictive of treatment nonadherence over and above the influence of guidance, which was added as dummy-coded variables (content-focused guidance vs administrative guidance; adherence-focused guidance vs administrative guidance) as a third step into the model. The final model explained 9.4% of the variance in treatment nonadherence (∆ *R*^2=^.05, *P<*.001; *t*_385_=4.26, *P<*.001; *t*_385_=4.01, *P<*.001).

**Table 2:** Results of the imputed hierarchical multiple linear regression analysis to identify user characteristics predictive of treatment nonadherence over guidance formats.

Variable		*R*^2^ or ∆ *R*^2a^	B (SE)^b^	Beta^c^	*P*
**Step 1**		.02			.09
	Constant		3.22 (1.08)		
	Gender		0.53 (0.31)	.09	.09
	Education		0.47 (0.25)	.10	.06
	Age		–0.00 (0.01)	–.01	.79
**Step 2**		.02			.046
	Constant		0.28 (1.56)		.86
	Gender		0.42 (0.31)	.07	.17
	Education		0.44 (0.24)	.09	.07
	Age		–0.01 (0.01)	–.03	.58
	Stress		0.08 (0.04)	.14	.046
	Depression		–0.04 (0.02)	–.15	.04
	Emotional exhaustion		0.31 (0.21)	.09	.14
	Hope for improvement		0.26 (0.20)	.07	.20
**Step 3**		.05			<.001
	Constant		–0.49 (1.53)		
	Gender		0.32 (0.30)	.05	.29
	Education		0.44 (0.24)	.09	.06
	Age		–0.01 (0.01)	–.04	.39
	Stress		0.07 (0.04)	.12	.08
	Depression		–0.03 (0.02)	–.11	.13
	Emotional exhaustion		0.28 (0.20)	.08	.16
	Hope for improvement		0.36 (0.20)	.09	.07
	Guidance format (content-focused guidance vs administrative guidance)		1.31 (0.31)	.24	<.001
	Guidance format (adherence-focused guidance vs administrative guidance)		1.24 (0.31)	.23	<.001

^a^*R*^2^ is for step 1 and ∆ *R*^2^ for steps 2 and 3.

^b^Unstandardized regression coefficient and unstandardized standard error.

^c^Standardized regression coefficient.

### Interactions Between Guidance Formats and Predictors

Adding the interactions between the guidance formats and the other variables to the model did not significantly change explained variance (∆ *R*^2^=.02 *, P=*.95).

## Discussion

### Principal Results

The first aim of this study was to identify the adherence rates for an Internet-based mobile-supported stress management intervention. The content-focused guidance adherence rate was 71%, which is comparable to the rates found in other guided Internet-based stress management interventions (46%-88%) [21,22,83,84]. For the adherence-focused guidance and the administrative guidance conditions, we found adherence rates of 69% and 42%, respectively. Comparing adherence rates for adherence-focused guidance and administrative guidance to previous trials is difficult because most of those trials conducted in the field either used other guidance formats or did not report adherence rates. However, in a study with adherence reminders [85], a module completion rate of 35% was found, which is below the rate for adherence-focused guidance in this study (69%). Nevertheless, varying definitions and operationalization of module completion limit the comparability of treatment adherence rates between different studies. Despite the limited comparability, this study’s results suggest relatively high adherence rates for content-focused guidance and adherence-focused guidance and lower rates for administrative guidance.

Our second research goal was to investigate the influence of different guidance formats on adherence in an Internet-based mobile-supported stress management intervention. Similarly to studies on other target conditions, such as depression [31] and anxiety [38,56], this study suggests that participants show better adherence with guided treatments in comparison to unguided treatments. As predicted, content-focused guidance and adherence-focused guidance resulted in higher treatment adherence rates compared to unguided treatment with only administrative support (administrative guidance).

As hypothesized, both content-focused guidance and adherence-focused guidance have high adherence rates; therefore, the next step was to analyze their equivalence in terms of treatment adherence. For both guidance formats, adherence was equivalent. Despite the equivalence in adherence rates, both guidance formats differ in the amount of eCoaching each requires. Content-focused guidance included both reminders and written feedback from an eCoach on every completed module and required up to 4 hours of coaching time. In contrast, adherence-focused guidance consisted of adherence monitoring and feedback on demand and only required up to 1 hour of coaching time per participant during the intervention. Therefore, by choosing adherence-focused guidance regarding the costs of treatment, substantial savings may be made without a significant reduction in patient adherence. This finding is in line with the assumption that the active factor responsible for improving adherence in guided versus unguided self-help interventions is that the participant is accompanied through the intervention. Providing instructions or detailed feedback on the content the participants worked on within the modules seems less critical for continued participant engagement.

However, the incremental value of offering feedback on demand compared to only adherence monitoring from an eCoach remains yet unclear. Within the adherence-focused guidance concept, it is hypothesized that feedback on demand is an important component so that the eCoach is seen as having the participant’s best interests at heart. Offering support may be an antecedent for creating an adherence-promoting relationship and, according to the supportive accountability model [25], assumed to be necessary to maximize the effects of adherence monitoring. But, it is possible that the superiority of adherence-focused guidance compared to administrative guidance can be purely explained by the effect of personalized reminders. Thus, future studies should investigate whether having the possibility to contact an eCoach has an incremental influence on treatment adherence beyond adherence monitoring.

In the adherence-focused guidance study arm, monitoring adherence and sending a personalized reminder took up almost all the resources associated with this guidance format (approximately 1 hour per participant). In contrast, feedback on demand required much less resources. Hence, the question arises whether automatic reminders sent from the system on behalf of the eCoach have, in combination with feedback on demand, a similar effect on adherence, while requiring even less resources. Other studies have already shown positive effects on treatment adherence through automatic reminders [86].

If feedback on demand from a health professional is not a necessary component to achieve sufficient treatment adherence, adherence monitoring may also be performed by nonprofessionals. This would improve cost-effectiveness and dissemination. The eCoaches’ qualification level was not found to significantly influence treatment efficacy in Internet interventions for a range of conditions [27]. A study by Titov and colleagues [87] did not show significant differences in clinical efficacy between layperson- or clinician-assisted Internet interventions. Hence, the characteristics of an eCoach keeping participants adherent to an intervention should be further investigated. It also remains unclear whether a personal coach is necessary at all.

However, reducing human contact in Internet interventions can also entail potential risks for participants. Without content-related feedback, the eCoach may not become aware of problems participants may experience during the training. Thus, the risk for negative effects with Internet interventions for some individuals has the potential to be higher when receiving adherence-focused guidance instead of content-focused guidance. For this reason, negative effects should be investigated in future studies that compare different guidance formats [88,89].

Likewise, the guidance format could also influence the acceptance and attractiveness of Internet interventions, and thereby be important for dissemination. Therefore, varying guidance formats in Internet interventions should also be evaluated in terms of attractiveness and general acceptance [90,91].

The preceding discussion alludes to the many factors that can influence guidance in Internet interventions. Our third research question moves the focus from intervention characteristics to participant characteristics. It aimed to identify participant characteristics as predictors of nonadherence in an Internet-based mobile-supported stress management intervention. Although the guidance formats significantly predicted nonadherence in this study, the predictive value of the variables targeted in this analysis is small. The final model only explained 9.4% of the interindividual differences in nonadherence rates. Other variables, purported in previous literature as predictive of treatment nonadherence in Internet interventions, may prove to be incrementally important and should be investigated further. These include, for example, primary motivation, intention to adhere, and self-efficacy, as well as computer savviness, Internet affinity, and usability in addition to system- and program-related variables [25,44,92,93].

### Limitations

This study has several limitations. First, the participants were not randomized to the three study arms. Hence, the differences between the guidance formats may be confounded by other differences between the studies.

Second, participants in the adherence-focused guidance group showed a significantly higher percentage of female users at baseline compared to participants in the administrative guidance group. Furthermore, the content-focused guidance sample had a significantly higher education level compared to the administrative guidance and adherence-focused guidance samples. These unsystematic variations may have contributed to better adherence rates in the adherence-focused guidance and content-focused guidance groups compared to the administrative guidance group. But gender and education level were not significantly associated with treatment nonadherence. However, the total sample was found to be highly educated, which may limit the generalization to the population.

Third, the adherence rates identified in this study refer to an Internet-based mobile-supported stress management intervention administered in RCTs. Several studies indicate that adherence rates to Internet interventions in the context of RCTs are higher than those available from open-access websites [7,9,94]. Improved usage rates in trial participants may indicate a preference for formal structures (eg, assessments, contact with research staff), which are thought to promote adherence. In addition, the elaborated study inclusion process (ie, completion of two self-report assessments, sending of informed consent) may have led to the inclusion of more motivated individuals through self-selection. As a result, adherence rates may not apply to other Internet interventions or Internet interventions applied to a different context. Hence, treatment adherence rates in this study might not be generalizable to an Internet-based mobile-supported stress management intervention in an open-access practice. However, Internet interventions in open-access contexts lack the structure-giving elements of RCTs, such as contact with the study administration team. Hence, guidance could play an even more important role in open-access Internet interventions by structuring the course of treatment. Thus, the differences between content-focused guidance, adherence-focused guidance, and administrative guidance may have been underestimated in this study.

Fourth, generalizability of the treatment adherence rates in an Internet-based mobile-supported stress management intervention are further limited by only including employees with elevated stress levels in the studies. Thus, the findings of this study may not be relevant for settings with participants that were not preselected based on their stress level. However, the stress level was not significantly associated with lower adherence rates beyond the guidance formats.

Fifth, only one adherence measure was included in the analysis. Different adherence measures need to be used which capture the quality of engagement with an intervention to a greater extent, such as time on website, number of completed homework assignments, and diary entries [95].

Sixth, as in most predictor studies, the analyses in this study were exploratory without any presumptions about the relationship between the predictors and adherence in order to generate hypotheses [96].

### Conclusion and Future Research

This study has important implications for research and practice. Guidance with focus on treatment adherence has the potential to be helpful in keeping participants involved in the training and, at the same time, keeping coaching costs low. Evaluating the cost-effectiveness as well as the comparative efficacy of the different guidance formats should be a next step to further complement the findings of this study [40]. Moreover, low treatment adherence is not negative per se because participants might quit the intervention due to improvement or because it does not meet their needs. Therefore, it is important to identify the reasons for nonadherence qualitatively to assess the impact of nonadherence for the efficacy of an intervention. Future research should focus on developing a theoretical model to explain the mechanisms behind treatment nonadherence in Internet interventions and derive variables from existing theories. This information can be used as a framework for the construction of an algorithm that generates risk profiles for participants that may be more susceptible to nonadherence.

## Abbreviations

RCT: randomized controlled trial
